# Proteomic and Physiological Analyses Reveal Putrescine Responses in Roots of Cucumber Stressed by NaCl

**DOI:** 10.3389/fpls.2016.01035

**Published:** 2016-07-15

**Authors:** Yinghui Yuan, Min Zhong, Sheng Shu, Nanshan Du, Jin Sun, Shirong Guo

**Affiliations:** ^1^Key Laboratory of Southern Vegetable Crop Genetic Improvement, Ministry of Agriculture, College of Horticulture, Nanjing Agricultural UniversityNanjing, China; ^2^Suqian Academy of Protected Horticulture, Nanjing Agricultural UniversitySuqian, China

**Keywords:** *Cucumis sativus* L., metabolic processes, proteome, putrescine, root, salt stress

## Abstract

Soil salinity is a major environmental constraint that threatens agricultural productivity. Different strategies have been developed to improve crop salt tolerance, among which the effects of polyamines have been well-reported. To gain a better understanding of the cucumber (*Cucumis sativus* L.) responses to NaCl and unravel the underlying mechanism of exogenous putrescine (Put) alleviating salt-induced damage, comparative proteomic analysis was conducted on cucumber roots treated with NaCl, and/or Put for 7 days. The results showed that exogenous Put restored the root growth inhibited by NaCl. Sixty-two differentially expressed proteins implicated in various biological processes were successfully identified by MALDI-TOF/TOF MS. The four largest categories included proteins involved in defense response (24.2%), protein metabolism (24.2%), carbohydrate metabolism (19.4%), and amino acid metabolism (14.5%). Exogenous Put up-regulated most identified proteins involved in carbohydrate metabolism, implying an enhancement in energy generation. Proteins involved in defense response and protein metabolism were differently regulated by Put, which indicated the roles of Put in stress resistance and proteome rearrangement. Put also increased the abundance of proteins involved in amino acid metabolism. Meanwhile, physiological analysis showed that Put could further up-regulated the levels of free amino acids in salt stressed-roots. In addition, Put also improved endogenous polyamines contents by regulating the transcription levels of key enzymes in polyamine metabolism. Taken together, these results suggest that Put may alleviate NaCl-induced growth inhibition through degradation of misfolded/damaged proteins, activation of stress defense, and the promotion of carbohydrate metabolism to generate more energy.

## Introduction

Soil salinity is one of the most serious environmental constraints to farming production, owing to both natural reasons and inappropriate agricultural operations (Munns and Tester, 2008). Although variable strategies existed for dealing with salt stress, most crops could not grow well on saline soil. Salt tolerance is a complex trait, involved in osmotic adjustment, toxic ions exclusion, and compartmentalization, as well as morphological changes (Munns, 2005). Most studies focused on salt-induced responses in the shoot tissues, since minimizing toxic ions accumulation in leaf is essential for plant growth and productivity (Julkowska et al., 2014). However, root is the first organ exposed to salt stress, and sometimes even shows greater growth reduction than shoot (Bernstein et al., 2004; Contreras-Cornejo et al., 2014). Root growth inhibition is the most obvious change caused by salt stress. Arabidopsis root exposed to NaCl exhibited a reduced meristem size and smaller mature cells, thus resulting in primary root growth inhibition (West et al., 2004). Excessive salt ions accumulating in the soil exert osmotic pressure to roots, which leads to the inhibition of water absorption capacity, over-accumulation of reactive oxygen species (ROS; Jiang et al., 2016), and interruption of membranes (Gupta and Huang, 2014). In addition, large amount of Na^+^ entering into root cells is always accompanied by sustained leakage of cytosolic K^+^, resulting in severe ion imbalance and metabolic disorders (Witzel et al., 2009).

Polyamines (PAs) are biologically ubiquitous aliphatic amines in all living cells. In plants, PAs have been implicated in various processes about plant growth and development, and play essential roles in abiotic, and biotic stress responses (Alcázar et al., 2010; Gill and Tuteja, 2010). The tetraamine spermine (Spm), triamine spermidine (Spd), and their precursors, diamine putrescine (Put), are the most common PAs studied in plants. Apart from the free forms, PAs in plants also occurred as conjugates with small molecules such as hydroxycinnamic acids and phenolic, and bound to macromolecules like proteins, and nucleic acids (Quinet et al., 2010; Tiburcio et al., 2014). In most plant species, Put is synthesized either from L-Arginine or L-Ornithine by arginine decarboxylase (ADC), or ornithine decarboxylase (*ODC*; Dalton et al., 2016), and then converted to Spd and Spm by the action of spermidine synthase (SPDS) and spermine synthase (SPMS). S-adenosylmethionine decarboxylase (SAMDC) provides aminopropyl groups for the synthesis of Spd and Spm (Bagni and Tassoni, 2001; Fuell et al., 2010). Polyamines catabolic process is catalyzed by diamine oxidases (DAO) for Put and polyamine oxidases (PAO) for SPD/Spm. Polyamine oxidases also catalyze the back-conversion of Spm to Spd (Moschou et al., 2008). The mutual conversion among endogenous Put, Spd, and Spm makes it difficult to ascertain their individual functions (Mattoo et al., 2010).

Numerous studies using mutants, ansgenic gene lines, or exogenous application demonstrated the positive role of PAs in salt tolerance (Urano et al., 2004; Wi et al., 2006; Yamaguchi et al., 2006; Kamiab et al., 2014). PAs inhibit the opening and induce closure of stomata by regulating voltage-dependent inward K^+^ channel (Liu et al., 2000) or H_2_O_2_ signal (Konstantinos et al., 2010), thus restricting transpiration and reducing water loss. PAs also regulated ROS homeostasis during salt stress by activating antioxidant defense system and directly scavenging ${\text{O}}_{2}^{-}$ and ·OH (Li et al., 2014; Saha et al., 2015). Interactions of PAs with ion channels especially contributed to the ion homeostasis under salinity conditions (Pottosin et al., 2014). Apart from these physiological changes, PAs are also reported to interact with proteins, the final executants of life activities, to control Plant adaptations. Previous studies reported that PAs could bind to charged spots at protein interfaces and thus regulate protein function by modulating electrostatic protein–protein interactions (Berwanger et al., 2010). PAs covalent binding with proteins by transglutaminase (TGase) helps stabilizing cell structure and function (Campos et al., 2013). Comparative proteome could provide comprehensive analysis of plant responses to environmental stresses regulated by PAs (Witzel et al., 2009). Proteomic analysis conducted on cucumber leaves revealed that increased salt tolerance by Spd was attributed to the increased levels of proteins involved in protein biosynthesis, antioxidant defense reaction, and energy metabolism (Li et al., 2013). Shi et al. (2013) found that pre-treatment of Put, Spd, and Spm enhanced salt and drought tolerance of bermudagrass by regulating carbon fixation, electron transport, energy, and defense pathways, and also activiating accumulation of osmolytes. All PAs effectively depressed protein tyrosine nitration and carbonylation by eliminating reactive nitrogen and oxygen species in leaves of citrus exposed to salinity stress (Tanou et al., 2014).

Cucumber (*Cucumis sativus* L.) as world popular vegetable is sensitive to soil salinity. Studies about exogenous substances such as calcium (He et al., 2012), salicylic acid (Hao et al., 2012), 24-epibrassinolide (An et al., 2016), and Spd (Li et al., 2013) induced proteome alterations in cucumber have been well-reported. Our previous study also reported the photosynthetic associated proteins regulated by Put (Shu et al., 2015). Little information about Put regulating proteomic changes in roots of cucumber exposed to salt stress is available. In this present work, we conducted 2-dimensional gel electrophoresis to analysis the root proteins responding to Put under NaCl stress conditions. A comprehensive analysis among Put regulated proteins and related metabolic processes was then carried out to explore the mechanism of Put in alleviating the damage of salt stress.

## Materials and methods

### Plant material and treatment

Cucumber (*Cucumis sativus* L. cv. Jinyou No. 4) seeds were sown in quartz sand, cultured in a greenhouse, and transplanted to plastic containers at the leaf two stage as previously described (Yuan et al., 2014). After 2 days pre-culture, the seedlings were treated as follows: (a) control, seedlings grown in full-strength Hoagland nutrient solution; (b) Put, seedlings grown in full-strength Hoagland nutrient solution containing 0.8 mM Put; (c) NaCl, seedlings grown in full-strength Hoagland nutrient solution containing 75 mM NaCl; (d) NaCl + Put, seedlings grown in full-strength Hoagland nutrient solution containing 75 mM NaCl and 0.8 mM Put. The experiment was arranged in a randomized complete block design with three replicates per treatment, that is, each treatment included three containers of total 36 plants. The concentrations of NaCl and Put were selected on the basis of previous experiment (data not shown). All the nutrient solutions were renewed every 2 days.

### Root growth determination and viability staining

The length of primary root was measured every day to calculate the average increment during the whole treatment period. After 7 days of treatment, cucumber roots were cut off and photographed. Root cell viability was then assessed by fluorescein diacetate (FDA)–propidium iodide (PI) double staining method as described by Bose et al. (2014). Root segments, including the root tip (1 cm), were cut off and stained with 5 μg mL^−1^ FDA for 3 min followed by 3 μg mL^−1^ PI for 10 min. Then the stained roots were observed using a Leica DM2500 microscope (Leica Microsystems, Wetzlar, Germany). Excitation wavelengths of 488 and 594 nm were used for FDA and PI imaging, respectively. Images were acquired with a digital camera (Leica DFC495, Leica Microsystems) equipped with a LAS V3.8 (Leica Microsystems) software.

### Measurement of free amino acids levels

After 7 days of treatment, root samples were harvested, oven dried, and acid hydrolyzed to analyze free amino acids by an automatic amino acid analyzer (Hitachi L-8900, Tokyo, Japan) according to Norden et al. (2004). Dried samples of root were suspended in 6 M HCl and then hydrolyzed at 110°C for 24 h. The hydrolyzed samples were centrifuged at 3000 × g for 5 min, and the supernatant was collected and evaporated to dryness. The dried samples were then redissolved in 0.1 M sodium citrate buffer (pH 2.2). The resulting amino acids solution was analyzed with 17 kinds of L-amino acid (sigma) as the standard. Results were determined as mg per 100 mg of dry samples (% DW).

### Analysis of endogenous polyamines

Endogenous polyamines levels were analyzed by high-performance liquid chromatography (HPLC) according to Shu et al. (2015) with small modifications. After 7 days of treatment, root samples were harvested and homogenized in 5% (W/V) cold HClO_4_ and incubated on ice for 1 h. After centrifugation for 20 min at 12,000 × g, the supernatant was used to determine free, and conjugated polyamines and the pellet was used to determine bound polyamines. For conjugated and bound polyamines, samples were hydrolyzed at 110°C for 18 h in 6 M HCl and then evaporated at 70°C. The residue was re-suspended in 5% HClO_4_ and used to measure polyamines contents with the non-hydrolyzed supernatant. The resulting solutions were mixed with 2 M NaOH and benzoyl chloride, and vortexed vigorously. After incubation for 30 min at 37°C, saturated NaCl solution was added to terminate the reaction. The benzoyl polyamines were extracted with cold diethyl ether. Then the diethyl ether phase was evaporated to dryness and re-dissolved in 64% (v/v) cold methanol. Finally, polyamines were assayed by HPLC 1200 series system (Agilent Technologies, Santa Clara, CA) with a C18 reverse phase column (4.6 by 250 mm, 5 μm Kromasil) and a two solvent system including a methanol gradient (36–64%, v/v) at a flow rate of 0.8 mL min^−1^. Standard Put, Spd, and Spm (Sigma) were treated in similar way.

### Protein extraction and 2-dimensional gel electrophoresis (2D) analysis

After 7 days of treatment, total root proteins were extracted using a trichloroacetic acid (TCA)-acetone precipitation method modified from Hurkman and Tanaka (1986). Fresh root samples (2 g) were ground to fine powder with liquid nitrogen and resuspended in 6 mL of ice-cold extraction buffer which contains 20 mM Tris-HCl (pH 7.5), 1 mM ethylenebis(oxyethylenenitrilo)tetraacetic acid (EGTA), 1 mM dithiothreitol (DTT), and 1 mM phenylmethyl sulfonyl fluoride (PMSF). After standing on ice for 20 min, the homogenate was centrifuged at 15,000 × g at 4°C for 20 min. The resulting supernatant was transferred into a new centrifuge tube and precipitated with five volumes of ice-cold acetone containing 10% TCA and 0.07% β-mercaptoethanol at −20°C for at least 4 h. The resulting protein-containing suspension was centrifuged at 20,000 × g for 25 min. The protein pellet was washed three times with acetone containing 0.07% β-mercaptoethanol at −20°C for 2 h. The protein sample was air-dried and rehydrated in a rehydration buffer consisted of 7 M urea, 2 M thiourea, 4% 3-[(3-cholanidopropyl) dimethylammonio]-1-propanesulfonic acid (w/v), 40 mM DTT, 0.5% (v/v) immobilized pH gradient (IPG) buffer 4–7, and 0.01% (w/v) bromophenol blue. Protein concentrations were quantified using the Bradford method (Bradford, 1976). Bovine serum albumin (BSA) was used as the standard.

Isoelectric focusing (IEF) was performed with pH 4–7, 18 cm IPG linear gradient strips (GE Healthcare, USA). IPG strips were loaded with 350 μL of protein sample containing 800 μg protein in a rehydration tray for 12–16 h at 25°C. After rehydration, IEF was accomplished at 20°C on an Ettan IPGphor 3 (GE Healthcare, USA) with the following conditions: 100 V for 1 h, followed by 200 V for 1 h, 200 V for 1 h, 500 V for 1 h, 1000 V for 1 h, 4000 V for 1 h, a gradient of 10,000 V for 1 h, and then 10,000 V rapid focus, reaching a total of 75,000 V h. The electric current during IEF was no more than 50 μA per strip.

After running the first dimension, IEF strips were equilibrated in 2D equilibrium buffer [50 mM Tris–HCl, pH 8.8, 6 M urea, 30% glycerol (v/v), 2% sodium dodecyl sulfate (SDS)] containing 1% DTT for 15 min and then in the same equilibrium buffer containing 2.5% iodoacetamide for 15 min. Then the equilibrated strips were placed directly onto 12.5% polyacrylamide-SDS slab gels and sealed with 0.5% agarose solution containing bromophenol blue dye. The second dimensional SDS-polyacrylamide gel electrophoresis (SDS-PAGE) was conducted using the EttanDaltSix electrophoresis system (GE Healthcare, USA). Electrophoresis was carried out at 15 W per gel until the bromophenol blue dye front reached about 1 cm from the bottom of the gel. The resulting gels were stained with Coomassie Brilliant Blue (CBB) R-250 to visualize protein spots.

### Image and data analysis

After de-staining, images of CBB-stained 2-D gels were obtained using an Image scanner III (GE Healthcare, USA) and analyzed with Imagemaster 2D Platinum version 5.0 (GE Healthcare, USA). The abundance of each protein spot was estimated by the percentage volume (vol%), which was normalized as the ratio of the volume of a single spot to the whole set of spots present in the gel. Only spots with significant (Duncan's multiple range test at the *P* < 0.05 level) and reproducible changes (at least 1.5-folds change in abundance) in three replicates were used for mass spectrometry.

### Protein identification

Differentially expressed protein spots were excised from gels and in-gel protein digestion was carried out as described by He et al. (2012). The resulting peptides were used for MALDI-TOF/TOF analysis with an ABI 5800 Proteomics Analyzer MALDI-TOF/TOF analyzer (Applied Biosystems, Foster City, CA, USA). Spectral data were used to search NCBI (http://www.ncbi.nlm.nih.gov/) and cucumber genomics database (http://cucumber.genomics.org.cn) with the software MASCOT version 2.2 (Matrix Science, London, UK). MS + MS/MS spectra search criteria in the databases were: trypsin as enzyme, one missed cleavage site, carbamidomethyl set as fixed modification, methionine oxidation allowed as dynamical modification, peptide mass tolerance within 100 ppm, the fragment tolerance set to ± 0.4 Da, and minimum ion score confidence interval for MS + MS/MS data was set to 95%.

### Protein functional classification

The differentially expressed proteins were classified according to the biological processes in which they are involved based on UniProtKB (http://www.uniprot.org/) and KEGG (http://www.genome.jp/kegg/) databases. Hierarchical clustering of protein expression patterns was performed using Cluster software version 3.0. Input data was calculated by dividing spot abundance at NaCl and/or Put treatment by abundance of the same protein spot at control and log_2_ transformed. The resulting heat map was visualized by Java Treeview.

### Quantitative real-time PCR (qRT–PCR) analysis

Root tissues were harvested after 1, 3, 5, and 7 days of treatment and extracted for total RNA using Trizol reagent (Takara, Otsu, Japan). A first-strand cDNA fragment was then synthesized using a SuperScript First-strand Synthesis System for qRT-PCR. qRT-PCR was performed according to the instructions provided by the SYBR® Premix Ex Taq™ II (Tli RNaseH Plus) kit (Takara). A 20 μL reaction mixture containing 2 μL of cDNA (diluted 1:5), 10 μL of SYBR® Premix ExTaq™ II (2 ×), 0.8 μL of each specific primer, 0.4 μL of ROX reference dye, and 6 μL of ddH_2_O was run in triplicate on a StepOnePlus™ Real-Time PCR System (Applied Biosystems). Thermal cycling was initialized at 95°C for 5 min, followed by 40 cycles of 95°C for 15 s, 60°C for 1 min, and a final extension of 15 s at 95°C. Relative expression levels were calculated using the 2^−ΔΔC_T_^ method, where the C_T_-values obtained from the amplification plots for tested genes were normalized against that of the housekeeping gene (actin) and compared with the control. Base sequences of these tested genes were identified by searching the NCBI and cucumber genomics database. The gene specific primers were designed using Beacon Designer 7 (Premier Biosoft International, CA, USA) and all primer sequences were listed in Supplementary Table 1.

### Statistical analysis

All data were statistically analyzed using SAS 13.0 software (SAS Institute, Inc., Cary, NC, USA) by Duncan's multiple range test at *P* < 0.05 level of significance.

## Results

### Root growth

**Figure 1:**
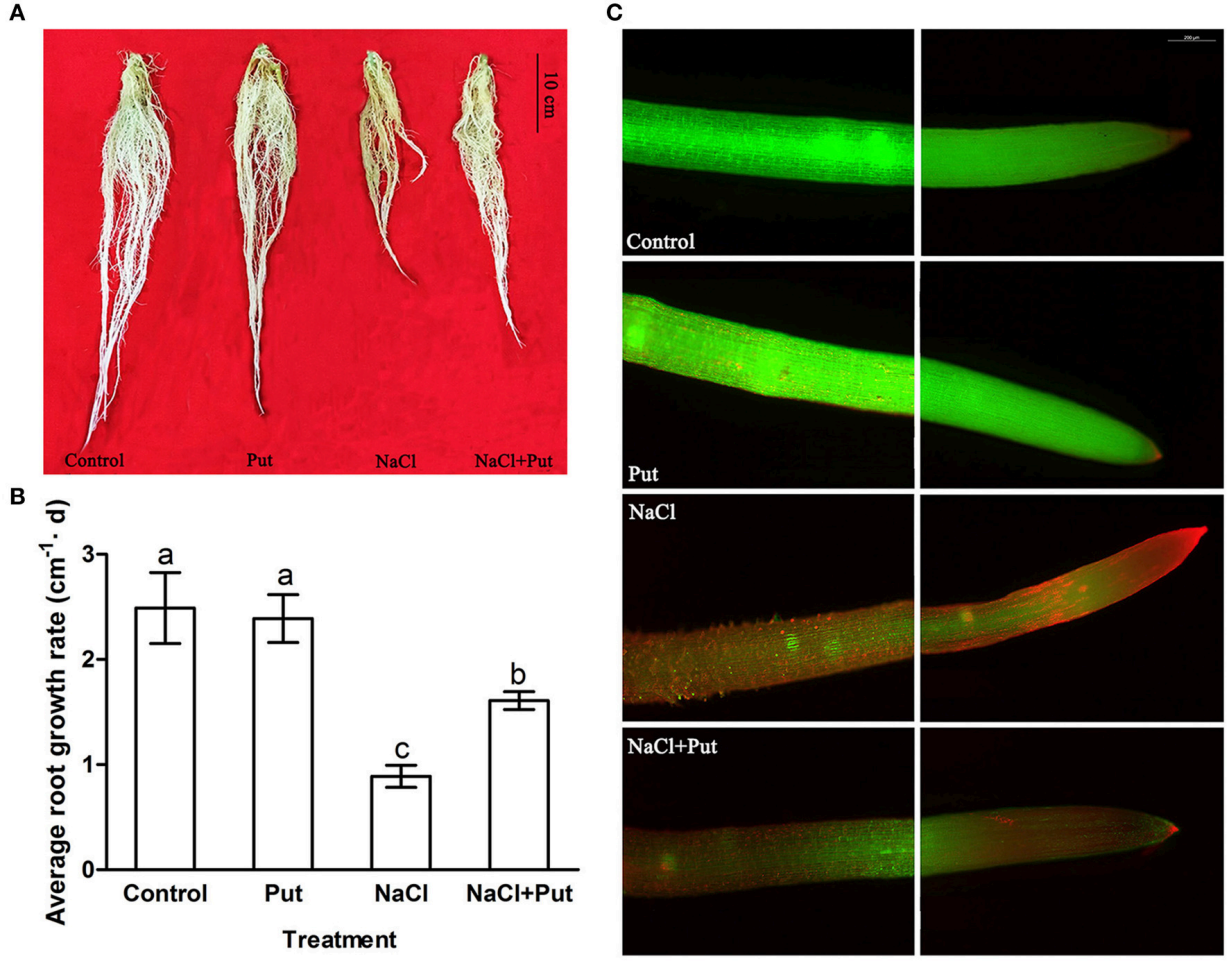
**Effects of exogenous putrescine (Put) on root morphology (A), average growth rate (B), and root viability (C) of cucumber seedlings in hydroponics with or without 75 mM NaCl for 7 d**. Each histogram represents a mean ± *SE* of six independent experiments (*n* = 6). Different letters indicate significant differences between treatments (*P* < 0.05) according to Duncan's multiple range tests. Control, seedlings cultured in normal nutrient solution; Put, seedlings cultured in nutrient solution with 0.8 mM Put; NaCl, seedlings cultured in nutrient solution supplemented with 75 mM NaCl; NaCl + Put: Both NaCl and Put added to the nutrient solution.

The effect of exogenous Put on root growth of cucumber seedlings exposed to NaCl stress was investigated (Figure 1). Seventy-five millimolars of NaCl treatment for 7 days seriously decreased cucumber root growth, and cucumber root gradually turned flaccid, filemot, and transparent (Figure 1A). Application of exogenous Put effectively alleviated the growth inhibition induced by NaCl stress, showing a 1.8-folds up-regulation in the average growth rate, but exerted no significant effect on roots of control plants (Figure 1B). Viability staining experiments also proved that Put reduced the root cells death under NaCl stress (Figure 1C), thus maintained the absorption of water and nutrients.

### Comparative proteomic analysis

**Figure 2:**
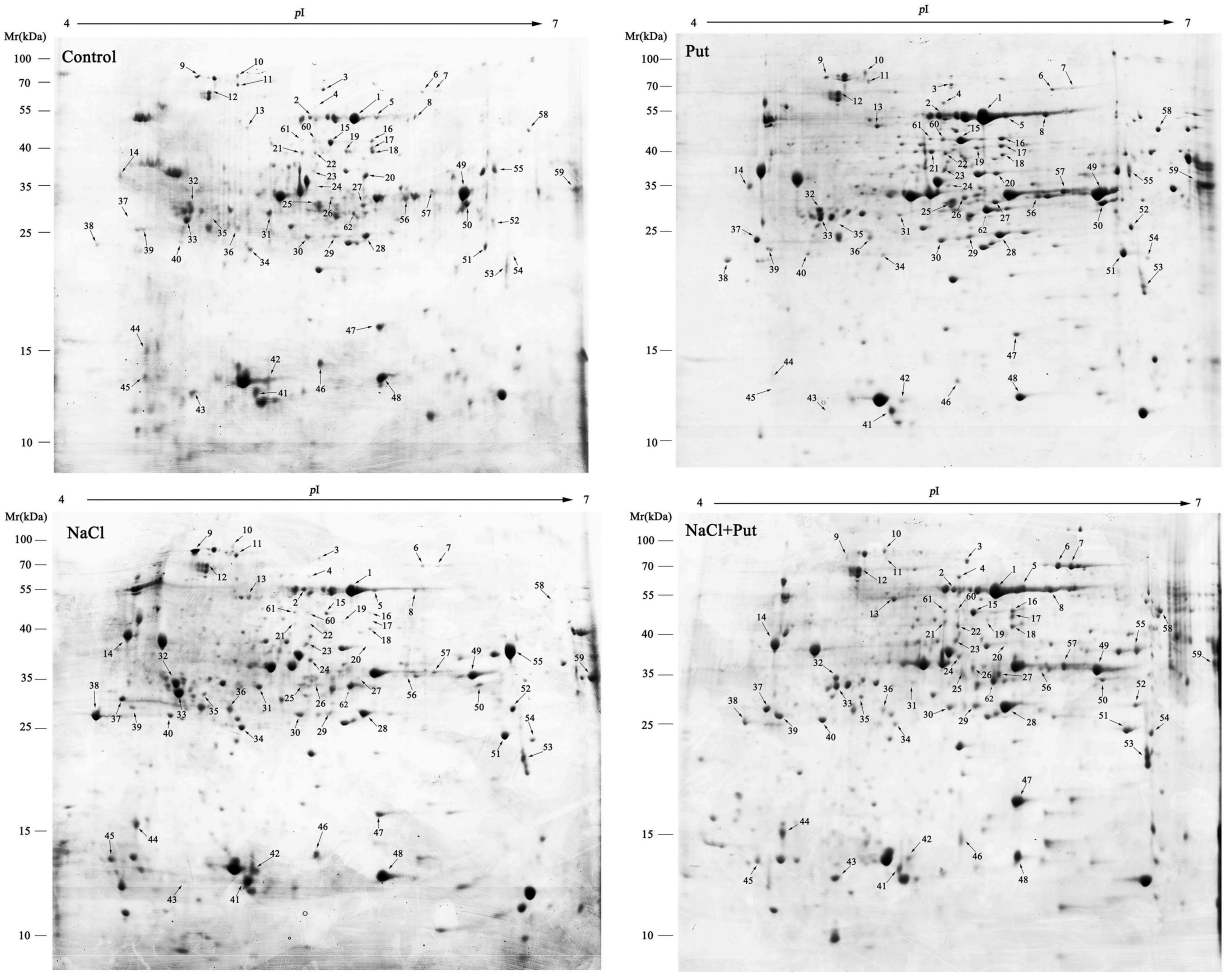
**Representative 2-DE gel images from root samples treated with NaCl and/or putrescine (Put)**. Total proteins were extracted and separated by IEF/SDS-PAGE then stained with Coomassie Brilliant Blue (R-250). An equal amount (800 μg) of total proteins was loaded onto 18 cm gel strip (pH 4–7, linear). The pI and molecular mass standards are indicated on the top and left side of each gel image. Sixty two differentially expressed protein spots are marked with arrows and numbers, and annotated according to the numbering in Table 1. Control, seedlings cultured in normal nutrient solution; Put, seedlings cultured in nutrient solution with 0.8 mM Put; NaCl, seedlings cultured in nutrient solution supplemented with 75 mM NaCl; NaCl+Put: Both NaCl and Put added to the nutrient solution.

A comparative analysis of proteome was conducted using root samples after 7 days of treatment to investigate the profiles of NaCl and/or Put-responsive, differentially expressed proteins. Approximately 400 reproducible protein spots were detected on the 2-DE gels (Figure 2; Supplementary Figure 1), of which 62 differentially expressed protein spots (changes ≥1.5 folds) were successfully identified by MALDI-TOF/TOF MS. These differentially expressed proteins were listed in Table 1.

**Table 1:** **List of differentially expressed proteins in response to NaCl and/or putrescine (Put)**.

**Spot no.^a^**	**Protein name**	**NCBI accession no**.	**Mr(kDa)/PI**		**MP^b^**	**Score**	**Cov^c^ (%)**	**Fold changes**			
			**Theoretical**	**Experimental**				**Control/Control**	**Put/Control**	**NaCl/Control**	**NaCl+Put/NaCl**
**CARBOHYDRATE METABOLISM (12)**											
**Glycolysis**											
1	Enolase isoform X1	XP_004143301.1	47.94/5.48	57/5.69	15	481	18.69	1.00	2.89	1.72	1.27
2	Enolase isoform X2	XP_011657684.1	43.13/5.8	57.4/5.45	14	318	45.98	1.00	4.93	1.63	1.25
5	Enolase isoform X1	XP_004143301.1	47.94/5.48	57.4/5.82	22	1100	71.62	1.00	3.02	1.68	2.58
6	2,3-Bisphosphoglycerate-independent phosphoglycerate mutase	XP_004147519.1	61.27/5.69	74.2/6.07	34	713	84.62	1.00	2.63	1.62	7.01
7	2,3-Bisphosphoglycerate-independent phosphoglycerate mutase	XP_004147519.1	61.27/5.69	74.2/6.15	34	986	86.05	1.00	1.54	1.82	11.03
59	Fructose-bisphosphate aldolase,cytoplasmic isozyme-like	XP_004143304.1	38.73/7.57	34.5/6.95	14	224	50.00	1.00	0.94	3.99	0.11
62	Probable fructokinase-4	XP_004145029.1	35.79/5.62	31.42/5.69	14	304	51.06	1.00	4.02	1.98	1.53
**Tricarboxylic Acid Cycle**											
49	Malate dehydrogenase, mitochondrial	XP_004137217.1	36.41/8.52	34/6.32	11	435	47.84	1.00	1.32	0.68	1.65
50	Malate dehydrogenase, chloroplastic	XP_011660098.1	43.65/8.11	32.6.34	16	354	45.50	1.00	0.65	0.31	1.26
56	Malate dehydrogenase, mitochondrial	XP_004137217.1	36.41/8.52	34/5.98	13	342	45.82	1.00	0.95	0.25	1.80
**Galactose Metabolism**											
8	Galactokinase	XP_011651088.1	55.21/5.69	57.4/6.08	24	535	55.91	1.00	4.26	2.86	2.49
**Sucrose Metabolism**											
10	Acid beta-fructofuranosidase-like	XP_004143623.1	69.80/4.92	93/5.06	16	588	30.63	1.00	1.16	0.57	1.89
**PROTEIN METABOLISM(15)**											
**Protein Folding and Assembly**											
3	Heat shock 70 kDa protein, mitochondrial	XP_004147511.1	73.25/5.69	79.6/5.52	32	435	60.59	1.00	0.72	0.46	2.43
4	Chaperonin CPN60-2, mitochondrial	XP_004147171.1	61.51/5.84	63.8/5.48	33	597	68.52	1.00	2.36	0.99	3.20
9	Heat shock protein 70	CAA52149.1	75.48/5.15	88.6/4.84	20	163	33.80	1.00	0.66	3.22	0.08
11	Heat shock protein 70	CAA52149.1	75.48/5.15	82/5.07	22	444	39.04	1.00	0.82	1.69	0.33
12	Protein disulfide-isomerase	KGN47715.1	57.27/4.88	70.5/4.91	27	1,080	70.39	1.00	1.55	0.57	3.07
**Protein Biosynthesis**											
23	Elongation factor 2	XP_011657107.1	95.03/5.97	38/5.47	19	340	27.52	1.00	3.03	1.86	1.42
35	Eukaryotic translation initiation factor 3 subunit J	XP_004140297.1	25.11/4.78	28.5/4.93	5	129	20.00	1.00	1.11	2.54	1.04
46	60S Acidic ribosomal protein P2-4	XP_004150488.1	11.42/4.53	13.5/5.52	9	631	81.58	1.00	0.34	1.09	0.61
**Protein Degradation**											
13	26S protease regulatory subunit 6A homolog	XP_004135596.1	47.81/4.96	52/5.12	17	130	46.57	1.00	3.16	1.90	2.10
36	Thiol protease aleurain-like	XP_004144033.1	39.55/6.26	26.25/5.04	10	489	43.25	1.00	1.49	2.41	0.67
40	Proteasome subunit alpha type-5	XP_011660309.1	26.10/4.75	24.33/4.73	11	342	64.56	1.00	0.58	1.65	2.02
**Protein Transport**											
32	Ran-binding protein 1 homolog a-like	XP_004145252.1	24.36/4.8	31.83/4.80	5	110	37.96	1.00	0.63	0.97	0.61
33	Ran-binding protein 1 homolog a-like	XP_011658664.1	22.41/5.06	29.5/4.78	12	551	68.02	1.00	0.82	1.66	0.56
39	Nascent polypeptide-associated complex subunit alpha-like protein 1	XP_004144316.2	21.90/4.40	25.33/4.51	11	378	75.50	1.00	1.28	1.69	4.06
**Protein Modification**											
42	Ubiquitin-conjugating enzyme E2 2	XP_004135191.1	17.45/5.4	12.5/5.21	2	149	17.76	1.00	0.32	1.00	0.44
**DEFENSE RESPONSE (15)**											
**Antioxidative Reaction**											
14	Peroxidase 2-like	XP_004142246.1	36.05/4.61	37.25/4.44	10	660	46.71	1.00	0.65	2.51	1.12
28	Ascorbate peroxidase, partial	AAQ88015.1	27.51/5.43	26.17/5.77	16	444	89.56	1.00	1.04	1.83	1.64
29	Ascorbate peroxidase	AGJ72850.1	27.56/5.44	25.75/5.59	11	259	52.61	1.00	1.79	1.00	1.86
30	L-ascorbate peroxidase, cytosolic-like	NP_001267635.1	27.55/5.43	25.5/5.44	17	251	71.49	1.00	1.39	1.77	1.05
34	Ascorbate peroxidase, partial	AAQ88015.1	27.51/5.43	24.25/5.12	13	300	62.25	1.00	0.62	1.57	0.44
45	Superoxide dismutase [Cu-Zn]-like isoform X1	XP_011658519.1	15.48/5.43	13/4.40	7	427	67.11	1.00	0.52	2.06	0.86
47	Superoxide dismutase [Cu-Zn],chloroplastic	XP_004145768.1	22.72/5.87	17/5.85	6	330	61.43	1.00	0.46	0.78	3.33
48	Peroxiredoxin-2B-like	XP_004142515.1	17.34/5.77	12.5/5.86	7	257	72.67	1.00	0.71	1.72	0.41
51	Glutathione S-transferase-like	XP_011659352.1	23.99/5.98	23.5/6.47	10	258	69.77	1.00	3.16	2.67	0.94
52	Glutathione S-transferase DHAR2	XP_011654937.1	23.90/6.18	27.6/6.51	13	521	63.85	1.00	1.28	1.79	0.75
55	Peroxidase 2-like	XP_004151583.1	37.68/5.64	37.25/6.51	7	227	34.01	1.00	0.92	4.92	0.23
57	Probable aldo-keto reductase 4	XP_004152112.2	37.89/5.78	35/6.13	13	319	42.61	1.00	1.57	0.55	3.38
**Other defense response**											
38	Chitinase	AAA33120.1	31.10/4.46	24/4.29	6	715	32.88	1.00	1.98	7.51	0.26
41	MLP-like protein 328	XP_004142283.1	17.37/5.08	12.5/5.16	10	403	82.12	1.00	0.76	1.53	0.46
43	Glycine-rich RNA-binding protein 3,mitochondrial-like	XP_004137435.1	17.25/7.82	12.25/4.81	7	278	57.31	1.00	1.32	0.22	7.36
**RESPIRATION (1)**											
53	Probable NAD(P)H dehydrogenase (quinone) FQR1-like 1	XP_004150766.1	21.73/6.43	21.25/5.58	9	713	51.72	1.00	1.32	1.88	1.12
**AMINO ACID METABOLISM (9)**											
15	S-adenosylmethionine synthase 2	XP_004153700.1	43.65/5.35	45.8/5.57	19	424	77.10	1.00	1.43	0.28	3.96
16	S-adenosylmethionine synthase 2	XP_004153700.1	43.65/5.35	45.75/5.80	13	145	40.71	1.00	2.80	0.29	5.45
17	S-adenosylmethionine synthase 2	XP_004153700.1	43.65/5.35	42.67/5.82	10	155	30.28	1.00	2.20	0.00	+
19	S-adenosylmethionine synthase 2	XP_004153700.1	43.65/5.35	42/5.65	11	119	31.30	1.00	1.24	0.32	1.41
21	S-adenosylmethionine synthase 2	XP_004153700.1	43.65/5.35	41.25/5.41	7	71	26.46	1.00	2.34	0.31	4.39
24	Arginase 1	XP_004145005.1	37.15/5.62	35.67/5.48	14	235	57.69	1.00	0.99	1.72	0.94
44	Glycine cleavage system H protein 2,mitochondrial	XP_004145438.1	6.93/4.98	14.75/4.52	4	203	47.10	1.00	3.49	1.53	1.11
60	S-adenosylmethionine synthase 2	XP_004135964.2	43.65/5.35	46.2/5.47	8	132	29.77	1.00	1.04	0.46	1.78
61	Fumarylacetoacetase	XP_004134793.1	47.60/5.21	46.4/5.40	13	88	42.33	1.00	0.96	0.54	1.72
**FATTY ACID METABOLISM (2)**											
25	Linoleate 13S-lipoxygenase 2-1,chloroplastic-like	XP_004142240.2	94.05/6.19	32.5/5.51	10	178	15.66	1.00	0.41	0.07	7.57
26	Enoyl-[acyl-carrier-protein] reductase [NADH],chloroplastic-like	XP_004142140.1	41.67/8.64	34/5.58	10	217	41.94	1.00	2.57	1.30	1.95
**SECONDARY METABOLISM (3)**											
20	Nitrile-specifier protein 5	XP_004139998.1	35.62/5.3	37.5.79	9	78	42.59	1.00	0.92	0.00	+
27	Nitrile-specifier protein 5	XP_004139998.1	35.62/5.3	32.75/5.74	13	108	43.52	1.00	2.08	0.39	4.38
54	Acylpyruvase FAHD1, mitochondrial	XP_004134224.1	23.64/6.21	23.2/6.61	8	639	64.55	1.00	0.91	1.66	1.43
**CELL RELATED PROTEIN (4)**											
18	Phragmoplast orienting kinesin 2	XP_011649250.1	158.81/5.09	40.25/5.80	30	69	26.35	1.00	0.99	0.42	3.29
22	Actin-7	XP_004147353.1	41.91/5.31	41.5/5.49	17	521	57.03	1.00	2.61	0.13	12.31
31	Tubulin beta chain-like	XP_004137743.1	50.73/4.73	30.25/5.22	9	87	28.03	1.00	0.37	0.84	0.27
58	Protein NETWORKED 1A	XP_011650959.1	211.48/5.18	48.25/6.68	39	75	22.04	1.00	3.43	0.75	5.96
**UNKNWON PROTEIN (1)**											
37	Uncharacterized protein At2g39795,mitochondrial-like	XP_004138451.1	28.75/4.69	26.33/4.46	5	232	27.67	1.00	2.98	2.43	1.23

a *Spot number corresponding with 2-DE gel as shown in Figure 2*.

b *Number of identified peptides*.

c *Percentage of sequence coverage by matched peptides*.

d *The values higher than 1.5 or lower than 0.67 indicate significant changes*.

**Figure 3:**
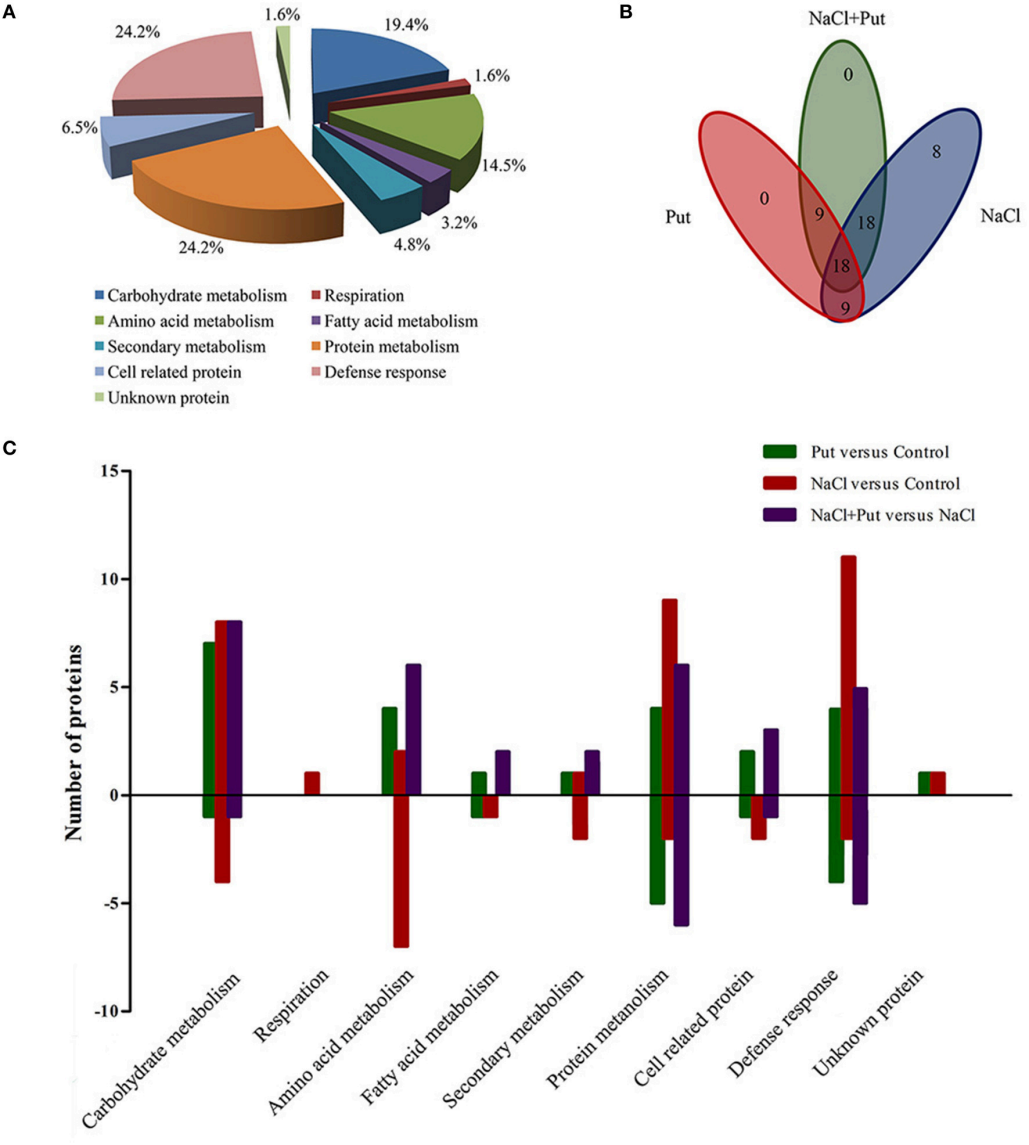
**Distribution of differentially expressed proteins by NaCl and/or putrescine (Put) in cucumber root. (A)** Functional classification of 62 differentially expressed proteins. **(B)** Venn diagram showing the number of overlapping proteins that were differentially regulated by Put, NaCl, and NaCl+Put as compared with the control. **(C)** The specific number of proteins with fold changes ≥1.5 (up-regulated, above the horizontal axis) or fold changes ≤ 0.67 (down-regulated, below the horizontal axis) regulated by Put, NaCl, and NaCl+Put compared with the control. Control, seedlings cultured in normal nutrient solution; Put, seedlings cultured in nutrient solution with 0.8 mM Put; NaCl, seedlings cultured in nutrient solution supplemented with 75 mM NaCl; NaCl+Put: Both NaCl and Put added to the nutrient solution.

All proteins respond to Put and/or NaCl were categorized into different classes according to their biological process (Figure 3A), among which one protein (spot 37) had no functional annotations in the database. The categories with a high level of expression variation are those involved in protein metabolism (24.2%), defense response (24.2%), carbohydrate metabolism (19.4%), and animo acid metabolism (14.5%). The number and overlapping of differentially expressed proteins were summarized in Figures 3B,C. Totally, 53 protein spots were significantly regulated by NaCl when compared with control (Figure 3B). Of those, 33 protein spots were up-regulated in abundance, and 20 protein spots were down-regulated. Most identified proteins involved in carbohydrate metabolism, protein metabolism, and defense response were up-regulated and most identified proteins involved in amino acid metabolism, fatty acid metabolism, and secondary metabolism were down-regulated (Figure 3C). However, 54 protein spots were significantly regulated by Put under control or NaCl stress conditions, of which 27 protein spots were regulated by Put under both control and NaCl stress conditions (Figure 3B). Put up-regulated the expression of most identified proteins involved in different biological processes, while also decreased the abundance of several proteins involved in defense response and protein metabolism (Figure 3C).

**Figure 4:**
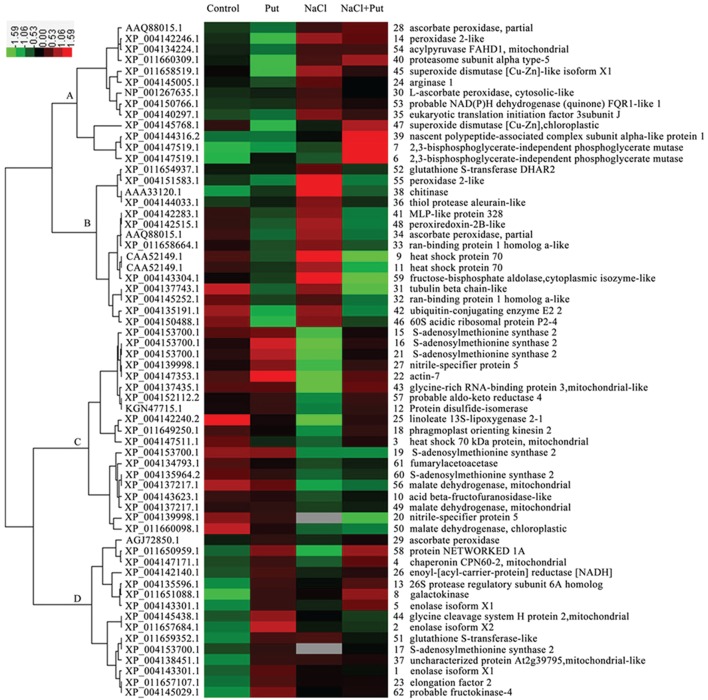
**Hierarchical clustering analysis of differentially regulated proteins in response to putrescine (Put) and/or NaCl**. The relative protein abundances were log_2_ transformed and imported to Cluster and Java Treeview to get the heat map. Columns represent treatments (Control, seedlings cultured in normal nutrient solution; Put, seedlings cultured in nutrient solution with 0.8 mM Put; NaCl, seedlings cultured in nutrient solution supplemented with 75 mM NaCl; NaCl+Put: Both NaCl and Put added to the nutrient solution). Each row represents individual protein spot with spot number and protein name labeled on the right side. Color scales of log_2_ values are shown, in which red and green show the higher and lower expression levels, respectively. And gray indicated protein missing under specific treatment.

In order to identify proteins with similar expression patterns, hierarchical clustering was performed (Figure 4). Cluster A was composed of 13 proteins that were up-regulated under both NaCl and NaCl+Put treatment as compared with those of the control. Most of these proteins were involved in defense response. Cluster B involved 15 proteins that were down-regulated by Put both under the control and NaCl stress conditions, and most of these proteins were corresponding to defense response and protein metabolism. Cluster C included 19 proteins that were down-regulated by NaCl, but recovered by the application of Put. Most identified proteins participating in amino acid metabolism were included in this cluster. Proteins involved in carbohydrate metabolism, especially tricarboxylic acid cycle, were also present in this cluster. Cluster D contained 15 proteins with increased abundance by Put under both the control and NaCl stress conditions. Most identified proteins functional in glycolysis (part of carbohydrate metabolism) were included in this cluster.

### Free amino acids contents

Many proteins related to protein and amino acid metabolism were identified in this study. Considering that amino acid is the raw material for protein synthesis, we measured the free amino acid levels under different treatment (Table 2). Glutamic acid (Glu) was the most abundant amino acid in cucumber root, occupied over 2% of dry weight, while cysteine, the least amino acid identified, occupied < 0.1% of root dry weight. Except for proline, all the other amino acids contents were increased by NaCl stress, leading to 15.2% increase in total amino acids level as compared with that of the control. Exogenous Put increased all amino acids levels in control plants, and total amino acids level was 1.23-fold higher than the control. Put applied to NaCl stressed plants further increased amino acids levels except for cysteine. Exogenous Put caused a higher increase in amino acids levels than NaCl, which reflected a promotion in amino acid metabolism and protein hydrolysis.

**Table 2:** **Effect of exogenous putrescine (Put) on free amino acid contents (%DW) in roots of cucumber seedlings exposed to 75 mM NaCl for 7 d**.

**Amino acid name**	**Treatment**			
	**Control**	**Put**	**NaCl**	**NaCl+Put**
Alanine	1.213 ± 0.012d	1.373 ± 0.007b	1.321 ± 0.005c	1.544 ± 0.004a
Arginine	0.992 ± 0.007c	1.232 ± 0.016a	1.125 ± 0.007b	1.232 ± 0.006a
Aspartic acid	1.645 ± 0.023d	2.070 ± 0.014a	1.917 ± 0.009c	1.995 ± 0.003b
Cysteine	0.064 ± 0.003c	0.078 ± 0.001b	0.089 ± 0.003a	0.079 ± 0.001b
Glutamic acid	2.262 ± 0.011d	2.623 ± 0.017b	2.532 ± 0.009c	2.793 ± 0.004a
Glycine	1.036 ± 0.008d	1.157 ± 0.008b	1.134 ± 0.004c	1.246 ± 0.002a
Histidine	0.367 ± 0.004c	0.433 ± 0.005a	0.404 ± 0.004b	0.436 ± 0.001a
Isoleucine	0.965 ± 0.008d	1.126 ± 0.005a	1.005 ± 0.005c	1.072 ± 0.001b
Leucine	1.605 ± 0.017d	1.847 ± 0.009a	1.681 ± 0.007c	1.774 ± 0.003b
Lysine	1.409 ± 0.015d	1.689 ± 0.011a	1.490 ± 0.006c	1.635 ± 0.004b
Methionine	0.274 ± 0.015b	0.283 ± 0.002ab	0.307 ± 0.002a	0.302 ± 0.002a
Phenylalanine	1.014 ± 0.008d	1.222 ± 0.010a	1.114 ± 0.004c	1.177 ± 0.003b
Proline	0.921 ± 0.009b	0.986 ± 0.007a	0.919 ± 0.003b	0.977 ± 0.005a
Serine	0.784 ± 0.007d	1.293 ± 0.016a	1.211 ± 0.004c	1.249 ± 0.003b
Threonine	0.453 ± 0.012c	0.921 ± 0.013ab	0.894 ± 0.005b	0.929 ± 0.002a
Tyrosine	0.305 ± 0.016b	0.614 ± 0.008a	0.605 ± 0.004a	0.614 ± 0.003a
Valine	1.238 ± 0.013d	1.437 ± 0.008a	1.320 ± 0.007c	1.409 ± 0.001b
Total amino acids	16.547 ± 0.134c	20.384 ± 0.154a	19.068 ± 0.073b	20.464 ± 0.035a

*Each value is the mean ± SE of three independent experiments (n = 3). Different letters indicate significant differences between treatments (P < 0.05) according to Duncan's multiple range tests. Control, seedlings cultured in normal nutrient solution; Put, seedlings cultured in nutrient solution with 0.8 mM Put; NaCl, seedlings cultured in nutrient solution supplemented with 75 mM NaCl; NaCl+Put: Both NaCl and Put added to the nutrient solution*.

### Endogenous polyamines metabolism

In response to NaCl stress, free, conjugated, and bound Put and Spm contents in cucumber roots were increased in comparison with those of the control (Table 3). Free Spd level was also increased by NaCl, whereas conjugated and bound forms of Spd were decreased significantly. Treatment with Put under NaCl stress condition caused increase in almost all kinds of PAs when compared with NaCl-only treatment, except for the bound Spm, which was decreased by Put. Exogenous Put also increased free PAs levels in control plants. Generally, NaCl stress caused increase in total Put and Spm levels, but decreased Spd level. Put treatment increased total Put and Spd contents both under control and stressed conditions, but decrease total Spm level stressed by NaCl.

**Table 3:** **Effect of exogenous putrescine (Put) on contents of endogenous free, conjugated, and bound PAs in roots of cucumber seedlings exposed to 75 mM NaCl for 7d**.

**Chemical**	**Treatments**	**Put** **(nmol·g^−1^ FW)**	**Spd** **(nmol·g^−1^ FW)**	**Spm** **(nmol·g^−1^ FW)**	**Put+Spd+Spm** **(nmol·g^−1^ FW)**
Free	Control	51.8 ± 4.08c	286.6 ± 24.17d	27.1 ± 1.49c	365.4 ± 21.89c
	Put	88.1 ± 4.17c	792.9 ± 20.95b	33.6 ± 2.88c	914.5 ± 16.91b
	NaCl	219.0 ± 18.29b	566.6 ± 25.77c	55.2 ± 6.31b	840.8 ± 7.67b
	NaCl+Put	301.4 ± 21.66a	1110.61 ± 53.79a	74.6 ± 6.04a	1486.6 ± 46.17a
Conjugated	Control	89.5 ± 7.82c	678.4 ± 18.30a	57.8 ± 7.92b	825.7 ± 16.88a
	Put	113.7 ± 7.10c	666.1 ± 54.01a	59.3 ± 8.35b	839.1 ± 62.34a
	NaCl	204.4 ± 8.50b	154.1 ± 12.69c	86.3 ± 3.51a	444.8 ± 18.68c
	NaCl+Put	300.2 ± 32.19a	270.4 ± 15.09b	102.2 ± 6.18a	672.8 ± 38.29b
Bound	Control	172.9 ± 14.32d	215.0 ± 7.67a	93.8 ± 3.90c	481.7 ± 19.68d
	Put	260.2 ± 10.89c	202.9 ± 10.94a	109.9 ± 9.91c	573.0 ± 19.71c
	NaCl	338.3 ± 12.60b	71.5 ± 3.97c	278.1 ± 7.96a	687.9 ± 7.48b
	NaCl+Put	419.9 ± 20.25a	120.8 ± 8.81b	201.5 ± 15.96b	742.2 ± 7.24a
Total	Control	314.1 ± 20.88d	1179.9 ± 45.18c	178.7 ± 10.88c	1672.8 ± 35.31d
	Put	461.9 ± 15.25c	1661.9 ± 33.30a	202.8 ± 6.12c	2326.6 ± 28.31b
	NaCl	761.7 ± 29.54b	792.2 ± 32.37d	419.7 ± 9.70a	1973.6 ± 24.95c
	NaCl+Put	1021.6 ± 36.37a	1501.9 ± 61.34b	378.3 ± 7.28b	2901.7 ± 60.51a

*Each value represents a mean ± SE of three independent experiments (n = 3). Different letters indicate significant differences between treatments (P < 0.05) according to Duncan's multiple range tests. Control, seedlings cultured in normal nutrient solution; Put, seedlings cultured in nutrient solution with 0.8 mM Put; NaCl, seedlings cultured in nutrient solution supplemented with 75 mM NaCl; NaCl+Put: Both NaCl and Put added to the nutrient solution*.

**Figure 5:**
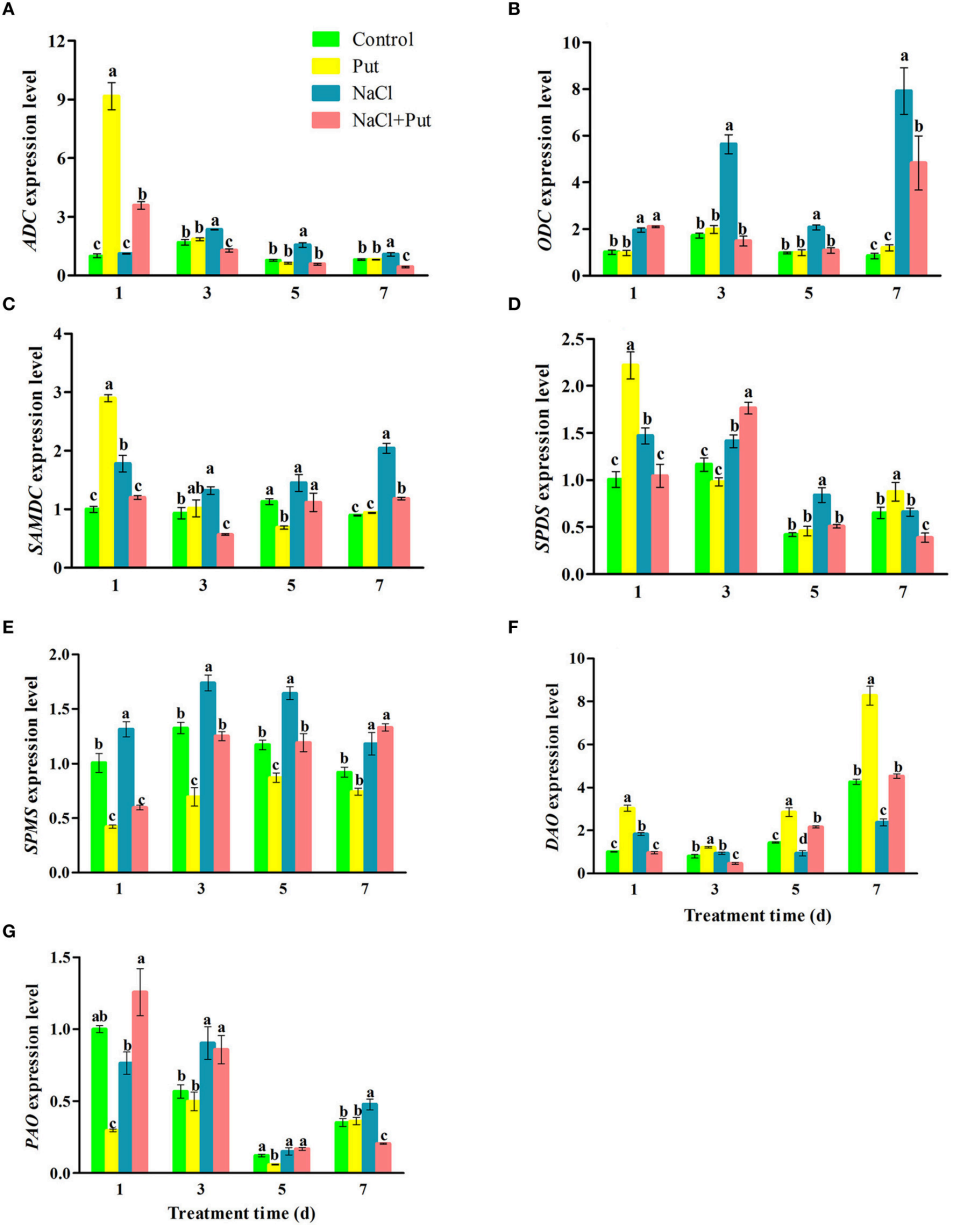
**Effects of exogenous putrescine (Put) on the expression of *ADC* (A), *ODC* (B), *SAMDC* (C), *SPDS* (D), *SPMS* (E), *DAO* (F), and *PAO* (G) in roots of cucumber seedlings exposed to 75 mM NaCl for 1, 3, 5, and 7d**. Each histogram represents a mean ± *SE* of three independent experiments (*n* = 3). Different letters indicate significant differences between treatments (*P* < 0.05) according to Duncan's multiple range tests. Control, seedlings cultured in normal nutrient solution; Put, seedlings cultured in nutrient solution with 0.8 mM Put; NaCl, seedlings cultured in nutrient solution supplemented with 75 mM NaCl; NaCl+Put: Both NaCl and Put added to the nutrient solution.

Expressions of seven genes encoding enzymes involved in PAs metabolism were then analyzed after 1, 3, 5, and 7 days of treatment. NaCl stress up-regulated all tested genes involved in PAs biosynthesis (Figures 5A–E). *PAO* expression was also increased by NaCl (Figure 5G), while *DAO* was first increased and then decreased after 5 days of NaCl treatment (Figure 5F). Put applied to NaCl-stressed plants down-regulated the expression of *ADC, ODC, SAMDC, SPDS*, and *SPMS* induced by NaCl, but increased *DAO* expression. After 1 day of treatment, Put up-regulated the expression of *ADC, SAMDC, SPDS*, and *DAO*, but down-regulated *SPMS* and *PAO* expression in the control plants. In fact, Put decreased the expression of *SPMS* and increased *DAO* expression in the control plants during the whole treatment period.

## Discussion

Multiple publications showed that polyamines can increase plants tolerance to various environmental stresses (Urano et al., 2004; Yamaguchi et al., 2006). In the present study, Put was found to restore cucumber root growth and root cell vitality decreased by NaCl stress (Figure 1). 2-DE analysis was conducted to obtain the profiling of the total proteins in cucumber roots responding to Put under NaCl stress conditions (Figure 2, Table 1). After quantitative and MALDI-TOF/TOF MS analysis, 62 differentially regulated protein spots were successfully identified. Proteins involved in protein metabolism, and defense response took up the highest percentage of differentially expressed proteins. Twelve carbohydrate metabolism related proteins and nine amino acid metabolism related proteins made the third and fourth group of identified proteins. In addition, several proteins participated in other metabolic processes were also identified. The regulation of Put and NaCl to these metabolic processes is discussed below.

### Putrescine regulates protein metabolism

Previous studies in bacteria provided direct evidence for the function of polyamines in protein synthesis (Algranati et al., 1977). Eukaryotic translation initiation factor 3 subunit J (eIF3J, spot 35) and elongation factor 2 (EF2, spot 23) are involved in the initiation and elongation stage of mRNA translation and protein synthesis (Kanhema et al., 2006). Put maintained the high level of eIF3J abundance induced by NaCl, and further increased EF2 expression in NaCl stressed conditions (Table 1, Figure 2), which proved the role of Put in protein synthesis. However, Put decreased the abundance of 60S acidic ribosomal protein P2-4 (RPP2D, spot 46), which is part of large subunit of ribosomes. This may be related to the function of Put in balancing ribosomal particles and protein synthesis, which was reported in *E. coli* by Algranati et al. (1977). Stimulating the synthesis of specific proteins by Put might play an important role in coping with salt stress.

Salt stress leads to protein unfolding or misfolding, which affects protein conformation and function, and causes metabolic disruption (Kumari et al., 2015). In this study, five spots were identified as protein chaperones (Table 1). Three out of the five spots represented heat shock 70 kDa protein (HSP70, spots 3, 9, and 11). HSP70 can be induced by various environmental stresses (Tomanek and Sanford, 2003), and this was further demonstrated in our study by 3.22- and 1.69-folds increases in abundance (spots 9 and 11) under NaCl stress as compared with that of the control. However, Put significantly decreased HSP70 (spots 9 and 11) expression both under control and NaCl stress conditions (Table 1). This result was consistent with Li et al. (2013), who found that Spd could down-regulated the expression of HSP70. Polyamines were reported to influence the DNA binding capacity of heat shock transcriptional factor HSF in rat cells and thus affecting the accumulation of HSP70 mRNA (Desiderio et al., 1999). Königshofer and Lechner (2002) reported that under heat stress conditions, polyamine metabolic status in tobacco, or alfalfa cells influence the HSP synthesis by affecting cell membranes integrity and properties. Mitochondrial chaperonin CPN60-2 (spot 4) belongs to HSP60 family, implicated in mitochondrial protein import, and macromolecular assembly (Tsugeki et al., 1992). In cucumber leaf, CPN60-2 was not affect by NaCl stress, but down-regulated by exogenous Spd (Li et al., 2013). In the present study, CPN60-2 in cucumber root was not changed under NaCl stress condition either, but significantly up-regulated by Put. Put also increased the abundance of protein disulfide-isomerase (PDI, spot 12), which catalyzes the formation, breakage or rearrangement of disulfide bonds during proteins folding (Gruber et al., 2006). Hence, Put may regulate chaperones to facilitate protein folding and prevent unfolding/misfolding-induced protein aggregation, thus reestablishing normal protein conformation and maintaining cellular metabolism when exposed to salt stress.

Votyakova et al. (1999) reported that proteins covalently attached with PAs became more resistant to proteolysis. Wajnberg and Fagan (1989) demonstrated that polyamines inhibited the ATP-dependent degradation of ubiquitinated proteins. Ubiquitin-conjugating enzyme E2 2 (UBC2, spot 42) participates in protein modification by catalyzing covalent attachment of ubiquitin to proteins. This process is involved in ubiquitin-mediated protein degradation by the 26S proteasome (Cui et al., 2012). In accordance, Put decreased the abundance of UBC2 under control and NaCl stress conditions (Table 1). This result suggested that Put not only targeting energy consumption (Wajnberg and Fagan, 1989) but also the ubiquitinated processes to inhibit protein degradation. However, Put further up-regulated the NaCl-induced increase in 26S protease regulatory subunit 6A homolog (RPT5A, spot 13) and proteasome subunit alpha type-5 (PSMA5, spot 4), which were also involved in the degradation of ubiquitinated proteins. Combination with the response in chaperones, Put may accelerate the degradation of misfolded/damaged proteins caused by NaCl to alleviate salt stress induced-damage. In addition, Put regulated protein metabolism may reprogram proteome of cucumber roots in response to salt stress, which is supported by Tanou et al. (2014), thus maintaining cell homeostasis.

### Putrescine activates stress defense responses to alleviate salt induced damage

Plants activate a broad array of defense responses to protect themselves from stress induced injuries. Chitinases are usually considered as pathogenesis-related proteins, and widely distributed in plants. Despite of their role against pathogen stress, chitinases are also implicated in various abiotic stresses (Grover, 2012). In the present work, Put increased chitinase (spot 38) abundance under control conditions (Table 1), indicated a promotion in plant defense. Glycine-rich RNA-binding protein (GR-RBP) has a role in RNA transcription or processing during stress by acting as RNA chaperone. GR-RBP7 was also reported to be involved in plant innate immunity (Lee et al., 2012). The increase in GR-RBP3 (spot 43) abundance induced by Put further proved the involvement of PAs in plant immunity. Apart from the plant immunity, redox regulation also represents essential defense response. In the current study, a total of 12 spots (spots 14, 28, 29, 30, 34, 45, 47, 48, 51, 52, 55, and 57) were identified as antioxidant related proteins and mostly showed up-regulated expression when exposed to NaCl stress (Table 1). The ability of Put in increasing the activities of antioxidant enzymes, such as superoxide dismutase (SOD), peroxidase (POD), and ascorbate peroxidase (APX), to alleviate oxidative damage caused by environmental stresses has been widely studied (Verma and Mishra, 2005). In this study, Put application to NaCl-stressed seedlings further improved the abundance of SOD (spots 45 and 47), POD (spot 14), APX (spots 28, 29 and 30), and glutathione S-transferases (GST, spot 51), which implied that Put regulated both abundance and activities of antioxidant enzymes to mitigate oxidative damage induced by salt stress. Aldo-keto reductase 4 (AKR, spot 57) belongs to AKR superfamily, which play central roles in response to oxidative stress by detoxifying toxic aldehydes, and ketones to the appropriate alcohol (Hyndman et al., 2003). AKR was also up-regulated by Put (Table 1). These results indicated that Put could enhance plant defense responses to reduce stress injuries and gain better growth in NaCl stress conditions.

### Putrescine promotes energy metabolism

Plants struggled in stress conditions need a substantial amount of energy to maintain their growth and development. Mitochondrial respiratory chain, the main provider of energy needed for cellular metabolism, has been reported to be activated by salt stress (Fry et al., 1986). Consistently, the abundance of NAD(P)H dehydrogenase (spot 53) was induced by NaCl in the present study, indicating an activation of respiratory electron transport systems. Carbohydrates metabolism, especially glycolysis (EMP), and tricarboxylic acid (TCA) cycle is also a primary source of energy for plant metabolism (Kumari et al., 2015). In the present study, seven differentially expressed spots were identified as enzymes participated in glycolysis (Table 1). Fructokinase-4 (FRK, spot 62), fructose-bisphosphate aldolase (FBA, spot 59), 2,3-bisphosphoglycerate-independent phosphoglycerate mutase (PGAM, spots 6 and 7), and enolase (spots 1, 2, and 5) were all up-regulated by NaCl, suggesting that roots attempted to generate more energy to cope with salt stress. However, NaCl stress decreased the abundance of malate dehydrogenase (MDH, spots 49, 50, and 56) involved in TCA cycle.

Several studies reported the involvement of PAs in adjusting EMP-TCA metabolism under stressed environment. Jia et al. (2010) found that exogenous Spd enhanced hypoxia stress tolerance by up-regulating activities of enzymes functioning in TCA cycle. Put sprayed to leaves partly reserved the NaCl-reduced activities of phosphofructokinase (PFK), pyruvate kinase (PK), and phosphoenolpyruvate pyruvate kinase (PEPC) involved in glycolysis metabolism, as well as activities of succinate dehydrogenase (SDH), isocitrate dehydrogenase (IDH), and MDH in TCA cycle (Zhong et al., 2015). Here we found that despite of decreased FBA abundance, exogenous Put increased the abundance of FRK, PGAM, and FBA both under control and NaCl stress conditions. What's more, Put partly reserved the MDH expression decreased by NaCl. All of these changes suggested that exogenous Put could promote EMP-TCA pathway activity. In addition to directly regulating these enzymes, Put may also affect TCA cycle through its metabolite, γ-aminobutyric acid (GABA; Gill and Tuteja, 2010). Adjusting EMP-TCA pathway to product more energy may be an important mechanism for Put to alleviate salt stress induced damage.

### Polyamines and amino acid metabolism play key roles in salt tolerance

**Figure 6:**
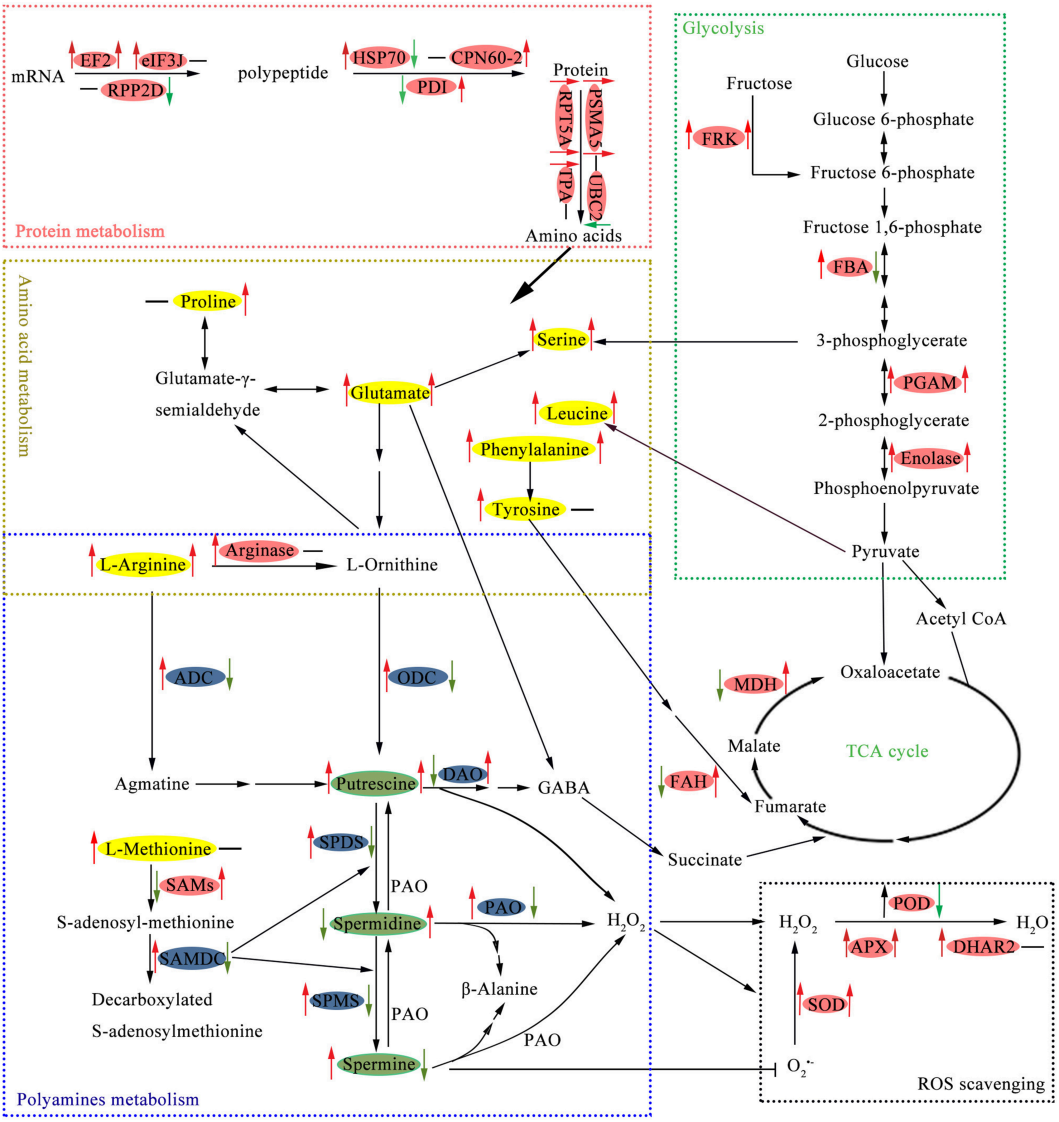
**Schematic presentation of main metabolic pathways regulated by putrescine (Put) in cucumber root exposed to NaCl**. Changes in protein abundance (marked in red ellipse), amino acids contents (yellow), PAs levels (green), and gene expression (blue) were integrated. Arrows on the life side of the ellipses indicate changes induced by NaCl as compared with the control, and arrows on the right side indicate changes induced by Put in NaCl stressed conditions. Red or green arrows represent up-regulation or down-regulation, respectively, and the black short lines indicate no change. APX, ascorbate peroxidase; CPN60-2, chaperonin CPN60-2; DHAR2, glutathione S-transferase DHAR2; EF2, elongation factor 2; eIF3J, eukaryotic translation initiation factor 3 subunit J; FAH, fumarylacetoacetase; FBA, fructose-bisphosphate aldolase; FRK, fructokinase; HSP70, heat shock 70 kDa protein; MDH, malate dehydrogenase; PDI, protein disulfide-isomerase; PGAM, 2,3-bisphosphoglycerate-independent phosphoglycerate mutase; POD, peroxidase; PSMA5, proteasome subunit alpha type-5; RPP2D, 60S acidic ribosomal protein P2-4; RPT5A, 26S protease regulatory subunit 6A homolog; SAMs, S-adenosylmethionine synthase; SOD, superoxide dismutase; TPA, thiol protease aleurain-like; UBC2, ubiquitin-conjugating enzyme E2 2.

Amino acid metabolic pathways as well as the protective metabolites produced constitute integral parts of the plant immune system (Zeier, 2013). In this study, nine spots were identified as proteins participated in amino acid metabolism (Table 1), while seven of these also related to PAs biosynthesis. Polyamines and amino acid metabolism play key roles in connecting C and N metabolism and several secondary metabolism pathways (Majumdar et al., 2016). We summarized the crossed metabolism connecting PAs, amino acids, and EMP-TCA cycle in Figure 6.

NaCl stress increased the soluble amino acid levels, which were further improved by Put (Table 2). Free amino acids can serve as osmolytes to regulate osmotic stress caused by salt, and their increases in response to NaCl and/or Put is largely attributed to protein hydrolysis (Yuan et al., 2012). This is further proved by the simultaneous up-regulation of glycine content (Table 2) and glycine cleavage system H protein 2 (spot 44) abundance (Table 1). Glu is the most abundant amino acid in cucumber root (Table 2), and is considered as the center of N metabolism in plants. Glu can be converted into ornithine (Orn), a non-protein amino acid present in low concentration in plants, which is an important precursor for Put (Majumdar et al., 2016). The increase in arginine (Arg) level, another precursor for Put, provided sufficient substrates for the synthesis of Put. In addition, Arg can be converted to Orn and urea via arginase (spot 24), which showed increased abundance under NaCl stress condition but did not respond to Put (Table 1). This result indicated that Put production through *ODC* might be enhanced by NaCl stress. The up-regulated expression of *ODC* also proved this assumption. Glu and Orn contribute to proline (Pro) synthesis by different routes. Salt stress induced Pro accumulation is an important mechanism for osmotic regulation. However, NaCl stress exerted no significant influence on Pro level in this study (Table 2). The most probable reason for this result was the substantial synthesis of Put consumed Arg and Orn thus limiting Glu and Orn flow into Pro. External Put inhibited the synthesis of more Put by regulating *ADC* and *ODC* expression, and consequently more N was available for Pro production. In this situation, the direct absorption of Put from nutrient solution may contribute large part of the increase in endogenous Put level (Shu et al., 2015).

Methionine (Met) is also related to PAs synthesis through conversion into S-adenosylmethionine (SAM). S-adenosylmethionine synthase (SAMs, spots 15, 16, 17, 19, 21, and 60) catalyzes the biosynthesis of SAM from Met and ATP, and serves as a common precursor for PAs and ethylene (Gong et al., 2014). Transgenic experiments proved that SAMs contributed to stress tolerance by increasing PAs accumulation (Qi et al., 2010; Gong et al., 2014). Put up-regulated SAMs abundance both under control and NaCl stress conditions (Table 1), and Spd showed the similar effect (Li et al., 2013). The increase in SAMs was partly responsible for the elevation in endogenous PAs levels (Table 3). Although SAMs was significantly reduced by NaCl, PAs levels were still increased due to the transcriptional activation of PAs biosynthesis (Figure 5).

The protective roles of PAs are partly attributed to their association with low molecular weight compounds and macromolecules, therefore the conjugated and bound PAs are essential for plant stress tolerance. Quinet et al. (2010) observed that the conjugated PAs pool was positively linked with rice salt tolerance. The conjugated PAs pool was reduced by NaCl in the present study due to a large decrease in conjugated Spd level (Table 3). Put application increased the conjugated and bound PAs levels, thus stabilizing the intercellular small molecules and macromolecules to resist salt stress. Exogenous Put induced the accumulation of endogenous Put, also elevated the expression of *DAO* (Figure 5F), which proved the transcriptional activation reported by Quinet et al. (2010). Interestingly, the oxidation of Put is accompanied by the production of H_2_O_2_, and GABA, and the latter is key component connecting PAs and amino acid metabolism (Majumdar et al., 2016). Another protein, fumarylacetoacetase (FAH, spot 61), is involved in the multiple steps synthesis of acetoacetate and fumarate from L-phenylalanine or L-tyrosine (Herrera et al., 2010). Fumarate happened to be an intermediate of TCA cycle (Figure 6). Put reversed NaCl reduced FAH abundance is an indirect evidence for its regulation to TCA cycle.

**Figure 7:**
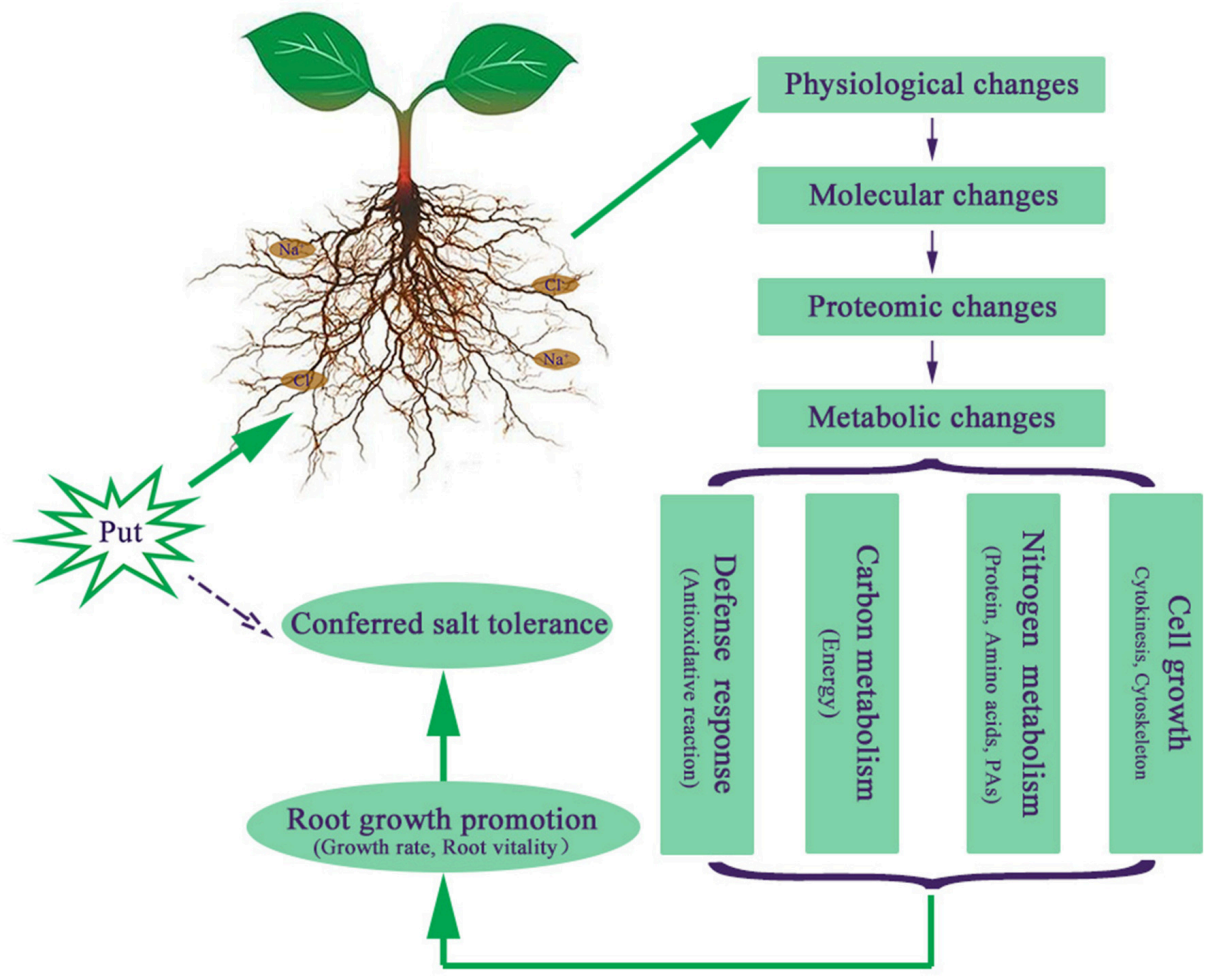
**Proposed model of the salt tolerance and root growth enhanced by putrescine (Put) in response to NaCl**.

Apart from the proteins mentioned above, Put was also found to regulate proteins involved in other metabolic pathways like lipid metabolism (spots 25 and 26) and nitrile formation (spots 20 and 27), indicating Put regulated various metabolic pathways to counteract salt stress-induced changes. Moreover, the identification of proteins related to cell division (spot 18) and cell cytoskeleton (spots 22, 31, and 58) proved the ability of Put to improve plant growth. We proposed a model of the NaCl stress tolerance conferred by external Put in cucumber seedlings (Figure 7). The application of Put to NaCl-stressed seedlings results in a series of responses from physiological level to metabolic level. Put enhanced stress defense capacity, regulated carbon and nitrogen metabolism to supply substrates and energy for various metabolic processes, and affected cell growth. All these changes contribute to the Put induced root growth promotion and salt tolerance.

## Conclusion

In conclusion, this work demonstrated that Put improving cucumber root growth and salt tolerance could be associated with the following points: (1) stimulating protein synthesis and degrading misfolded/damaged proteins induced by NaCl; (2) activation of stress defense response to alleviate salt induced injuries; and (3) providing more energy for various metabolic processes by up-regulating proteins involved in carbohydrate metabolism. This study provides comprehensive insights into the cell metabolism regulated by NaCl and/or Put, and would be able to better enrich our understanding of the mechanism by which Put improves the salt tolerance of cucumber seedlings.

## Author contributions

SG designed the study and guided the research. YY wrote the main manuscript text and performed the experiments. MZ and ND prepared all the figures and performed some of the experiments. SS and JS modified this manuscript. All authors reviewed and approved the manuscript.

## Funding

This work was supported financially by the National Natural Science Foundation of China (31401919, 31471869, and 31272209), the China Earmarked Fund for Modern Agro-industry Technology Research System (CARS-25-C-03), the Priority Academic Program Development (PAPD) of Jiangsu Higher Education Institutions, and the Research Fund for the Doctoral Program of Higher Education (20130097120015).

### Conflict of interest statement

The authors declare that the research was conducted in the absence of any commercial or financial relationships that could be construed as a potential conflict of interest.
