# Supplementary material for: Proteomic and Physiological Analyses Reveal Putrescine Responses in Roots of Cucumber Stressed by NaCl
Source: Front Plant Sci. 2016 Jul 15;7:1035. doi: 10.3389/fpls.2016.01035 (PMC4945654; doi:10.3389/fpls.2016.01035)
Supplement: Supplementary file 1 [file Table1.DOC]

*Supplementary Material*

# Proteomic and Physiological Analyses Reveal the Metabolic Processes Regulated by Putrescine in Roots of Cucumber (*Cucumis sativus* L.) Stressed by NaCl

Yinghui Yuan^1^, Min Zhong^1^, Sheng Shu^1^, Nanshan Du^1^, Jin Sun^1,2^, Shirong Guo^1,2*^

**^*^ Correspondence:**Shirong Guosrguo@njau.edu.cn

**Supplementary Table 1** Primers used for qRT-PCR analysis and their target genes.

| Gene | Primer sequence 5′ to 3′ | |
| --- | --- | --- |
| *ADC* | S | TGGCGAGAAAGGAAAGTT |
|  | AS | CATCAGCGAGCAAAGCAG |
| *ODC* | S | TAAATCGGCTGAACTCTC |
|  | AS | CAATGGCGGCTGAATA |
| *SAMDC* | S | GATTGTGAGGGTGCTG |
|  | AS | CGACCTTGGAGATGAG |
| *SPDS* | S | GCCGCCAGATATTCAA |
|  | AS | TGTCAATGCCGTTCAC |
| *SPMS* | S | ATGACGGAAAGAAAAGGGT |
|  | AS | ATTGAACAGAGGGGCAGAT |
| *DAO* | S | CGTTGTTAGCCGAGAAT |
|  | AS | GGTAGGTGAGGAAGTGA |
| *PAO* | S | TGGCATCCTGAACTTG |
|  | AS | GGTGAATCTGGCTGAG |
| *actin* | S | CAGGAATCCACGAAACTACT |
|  | AS | AGACCCTCCAATCCAAACAC |

*Note: ADC*, arginine decarboxylase; *ODC*, ornithine decarboxylase; *SAMDC*, S-adenosylmethionine decarboxylase; *SPDS*, spermidine synthase; *SPMS*, spermine synthase; *DAO*, diamine oxidases; *PAO*, polyamine oxidases; *actin*, housekeeping gene.
