# Supplementary figures and images for: Proteomic and Physiological Analyses Reveal Putrescine Responses in Roots of Cucumber Stressed by NaCl
Source: Front Plant Sci. 2016 Jul 15;7:1035. doi: 10.3389/fpls.2016.01035 (PMC4945654; doi:10.3389/fpls.2016.01035)

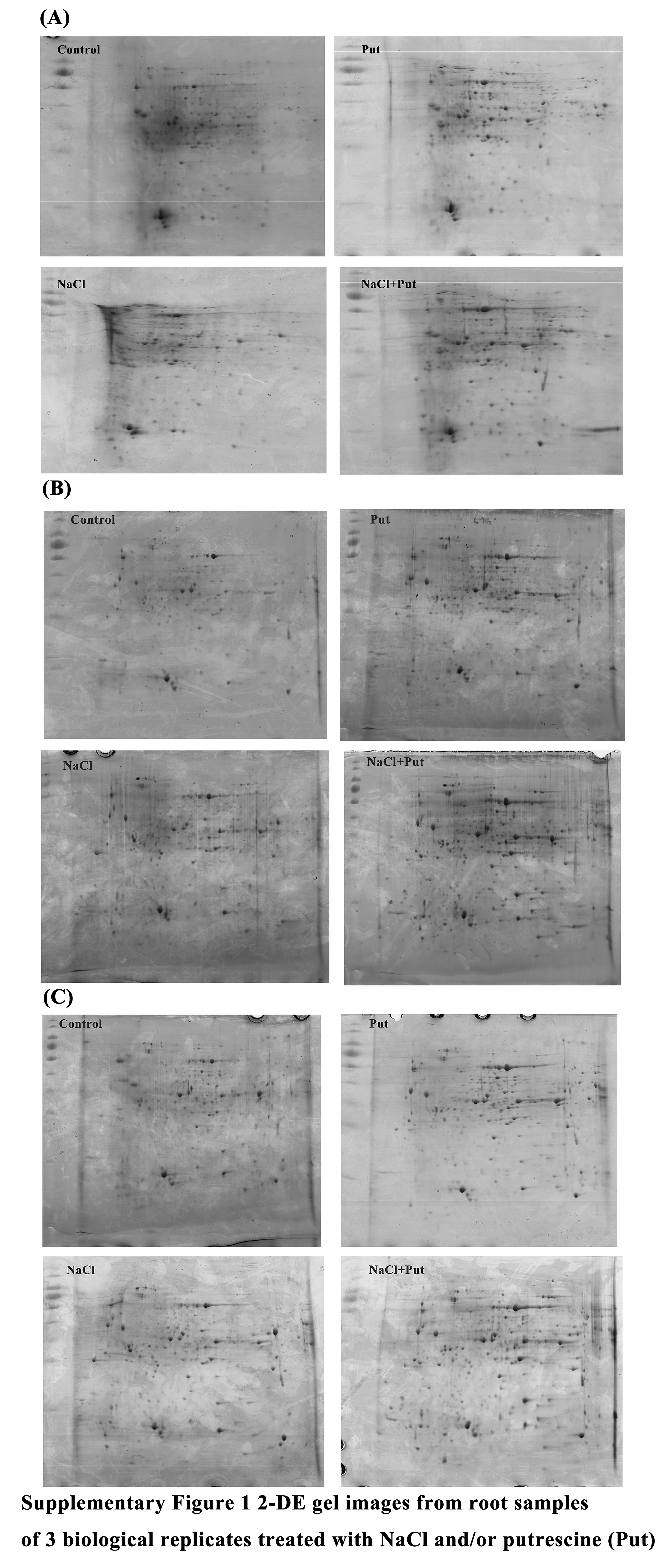

Supplement: Supplementary file 2 [file Image1.JPEG]
